# Supplementary material for: End Sequence Analysis Toolkit (ESAT) expands the extractable information from single-cell RNA-seq data
Source: Genome Res. 2016 Oct;26(10):1397–410. doi: 10.1101/gr.207902.116 (PMC5052061; doi:10.1101/gr.207902.116)
Supplement: Supplemental Material [file supp_gr.207902.116_Supp_Materials.tar.gz › ESAT-esat_v0.1/doc/html/class_jama_1_1_cholesky_decomposition.html]

ESAT: Jama.CholeskyDecomposition Class Reference


ESAT


- Main Page
- Related Pages
- Packages
- Classes
- Files

- Class List
- Class Index
- Class Hierarchy
- Class Members

All Classes Namespaces Files Functions Variables Enumerator Pages

- Jama
- CholeskyDecomposition

Public Member Functions |
List of all members

Jama.CholeskyDecomposition Class Reference

Inheritance diagram for Jama.CholeskyDecomposition:

[legend]

Collaboration diagram for Jama.CholeskyDecomposition:

[legend]

|  |  |
| --- | --- |
| Public Member Functions | |
|  | CholeskyDecomposition (Matrix Arg) |
|  | |
| boolean | isSPD () |
|  | |
| Matrix | getL () |
|  | |
| Matrix | solve (Matrix B) |
|  | |

## Detailed Description

Cholesky Decomposition.

For a symmetric, positive definite matrix A, the Cholesky decomposition is an lower triangular matrix L so that A = L\*L'.

If the matrix is not symmetric or positive definite, the constructor returns a partial decomposition and sets an internal flag that may be queried by the isSPD() method.

## Constructor & Destructor Documentation

|  |  |  |  |  |  |
| --- | --- | --- | --- | --- | --- |
| Jama.CholeskyDecomposition.CholeskyDecomposition | ( | Matrix | *Arg* | ) |  |

Cholesky algorithm for symmetric and positive definite matrix.

Parameters
:   |  |  |
    | --- | --- |
    | A | Square, symmetric matrix. |

Returns
:   Structure to access L and isspd flag.

## Member Function Documentation

|  |  |  |  |  |
| --- | --- | --- | --- | --- |
| Matrix Jama.CholeskyDecomposition.getL | ( |  | ) |  |

Return triangular factor.

Returns
:   L

|  |  |  |  |  |
| --- | --- | --- | --- | --- |
| boolean Jama.CholeskyDecomposition.isSPD | ( |  | ) |  |

Is the matrix symmetric and positive definite?

Returns
:   true if A is symmetric and positive definite.

|  |  |  |  |  |  |
| --- | --- | --- | --- | --- | --- |
| Matrix Jama.CholeskyDecomposition.solve | ( | Matrix | *B* | ) |  |

Solve A\*X = B

Parameters
:   |  |  |
    | --- | --- |
    | B | A Matrix with as many rows as A and any number of columns. |

Returns
:   X so that L\*L'\*X = B

Exceptions
:   |  |  |
    | --- | --- |
    | IllegalArgumentException | Matrix row dimensions must agree. |
    | RuntimeException | Matrix is not symmetric positive definite. |

Here is the call graph for this function:

---

The documentation for this class was generated from the following file:

- java/Jama/CholeskyDecomposition.java


---

Generated on Fri Aug 15 2014 15:11:06 for ESAT by  

 1.8.7
